# ESMR: a process framework for calibration dynamics across psychosomatic and psychiatric phenomena

**DOI:** 10.3389/fpsyt.2026.1832075

**Published:** 2026-05-21

**Authors:** Daisuke Yamashita

**Affiliations:** Department of Pediatrics and Child Health, Kurume University School of Medicine, Kurume, Fukuoka, Japan

**Keywords:** active inference, ESMR, metacognition, psychological therapy, psychosomatic medicine, reality grounding, recalibrability, transdiagnostic psychopathology

## Abstract

The brain can be modeled as a generative system that predicts bodily and environmental input and reduces mismatch through updating and action. From this perspective, stress is treated not as prediction error per se, but as a condition in which mismatch persists without sufficient recalibration. Health is therefore defined not as the absence of symptoms, but as the preservation of recalibrability, whereas psychopathology is conceptualized as the progressive fixation of recalibration failure. This paper proposes ESMR as a four-layer, two-timescale process framework for comparing psychosomatic conditions, psychiatric disorders, and psychosis-level phenomena in terms of partially shared failures of recalibration rather than as a single disease continuum. ESMR distinguishes E (Embodied Constraints), S (Salience Calibration), M (Metacognitive Model Revision), and R (Reality-Grounding Interface) across learning (L) and development (D). Development is treated not merely as slower accumulation of prior learning, but as change in the biological, representational, and self-regulatory architecture that alters both what forms of recalibration are possible and the background conditions under which learning can be taken up. A central distinction is that M concerns whether corrective information can be converted into updating, whereas R concerns whether already understood correction can be adopted as self-relevant reality under embodied, practical, and interpersonal constraints, with downstream transfer treated as a consequence rather than part of the R core. The framework predicts layer-specific cross-signatures rather than a single undifferentiated burden effect and outlines a staged empirical program using framing, disconfirmation, reality-grounding, and embodied/interoceptive tasks. ESMR is offered as a revisable, clinically oriented intermediate framework rather than a completed computational model.

## 1 Introduction

### 1.1 Predictive processing and calibration

The brain functions as a generative model that predicts upcoming sensory input based on internal models built from past experience (1–4). The discrepancy between prediction and actual input is prediction error, and the brain minimizes that error either by revising beliefs or by changing behavior. Active inference extends this framework to incorporate action and policy selection (1, 5–8). Prediction error is weighted by precision, and the brain dynamically adjusts the importance of information according to context (9–16).

### 1.2 Prediction mismatch generates stress

From a predictive-processing perspective, stress is not a mismatch per se, but a state in which mismatch persists because the system fails to recalibrate, update, or reorganize behavior accordingly (10). Dangerous stimuli, bodily discomfort, interpersonal conflict, and self-world incongruence all manifest as discrepancies between prediction and reality. If such discrepancies are temporary and can be used as material for recalibration, they are not pathological and may even become opportunities for learning. The problem arises when the discrepancy recurs, remains unresolved, and elicits the repeated use of fixed solutions. For this reason, the response to a threat is not inherently pathological. In objectively dangerous situations, a heightened response may be adaptive. Pathology emerges when the system cannot exit threat mode even under low-threat conditions and the internal model no longer updates in accordance with reality.

### 1.3 What is being “calibrated”?

Within this framework, calibration refers to preserving model revisability under embodied, practical, and interpersonal constraints, thereby maintaining coherence among prediction, attention, policy selection, and meaning-making. Recalibrability refers to the capacity to continue updating the internal model flexibly in response to new information. Here, calibration is not used merely as a synonym for expected free energy; instead, it functions as an operational concept for describing whether revisability is preserved under real-world constraints.

Calibration is operationally decomposed into four levels.

Embodied Constraints layer (E layer): calibrates biologically structured bodily input conditions, stimulus thresholds, gain, fidelity, and sensory resolution, thereby shaping what bodily perturbations are detectable, ambiguous, amplified, or constrained and setting the informational resolution on which subsequent salience assignment and learning depend.

Salience Calibration layer (S layer): governs significance assignment and precision weighting, including how bodily and environmental signals are foregrounded as threatening, safe, urgent, or otherwise learning-relevant before they are stabilized as explicit interpretations, beliefs, or policies.

Metacognitive Model Revision layer (M layer): governs perspective shifting, comparative revision, and the extent to which corrective information can be converted into updating of beliefs and behavioral policies; in humans, this capacity expands markedly with the development of language, abstraction, and self-referential reflection.

Reality-Grounding Interface layer (R layer): governs whether revised understanding can be adopted as self-relevant reality under embodied, practical, and interpersonal constraints, with downstream consequences for self-understanding, action, and acceptance.

These four levels correspond to the four layers of ESMR proposed in this paper: E, S, M, and R.

Calibration in humans cannot be understood apart from the biological and developmental architecture within which it occurs. What can be learned, how correction can be represented, and how durable that correction can become are not constant across the lifespan. They depend on the maturational state of bodily systems, the availability of language and symbolic mediation, the development of abstraction and metacognition, and later-age changes in plasticity and bodily constraints. For this reason, ESMR is intended not as a purely computational scheme, but as a biologically constrained framework for describing how human health and psychopathology emerge through structured patterns of learning, fixation, and recalibration over time.

From this standpoint, health should not be understood as a static equilibrium or a final error-free state. It is better conceived as a dynamic equilibrium in which recalibration remains possible over time despite continuing perturbation, biological change, and social demand. Pathology, in turn, is not mere dysfunction in the abstract, but the progressive fixation of once-functional response tendencies into increasingly rigid equilibria that preserve local viability at the cost of broader revisability.

### 1.4 The spectrum of psychopathology as failure of calibration

Stress is a state in which calibration has not occurred, whereas pathology is a state in which that failure has become fixed. From this perspective, phenomena ranging from health to psychosis can be understood as a spectrum defined by the depth and layer of calibration failure.

In health, recalibration occurs even when prediction error arises. In states of stress, recalibration is preserved but the load is high. In psychosomatic states, fixation is organized primarily through bodily and autonomic channels and often appears as the coupling of hyperarousal in the E layer with threat-biased salience allocation in the S layer. In psychiatric states, fixation extends into metacognitive revision and reality-grounded adoption. In psychosis-level states, reality-grounded adoption may diverge substantially from real-world constraints.

These are not sharply separated categories. Rather, they form a continuum that reflects differences in the depth, layer, and persistence of calibration failure (see Fixation of calibration: a spectrum from health to psychosis for details).

### 1.5 What is health? A link to salutogenesis

Health is not the absence of symptoms; it is a state in which recalibrability is preserved.

This perspective resonates strongly with Antonovsky’s salutogenic model of health (17). Antonovsky did not ask why people become ill, but why people remain healthy. He identified a common feature among those who maintain health: a sense of coherence (SOC), consisting of three components.

Comprehensibility: the sense that incoming stimuli and experiences can be understood in a meaningful way.

Manageability: the sense that resources are available to cope with them.

Meaningfulness: the sense that coping is worth the effort.

In ESMR terms, comprehensibility corresponds to being able to read signals from the body and the situation and understand what is happening; manageability corresponds to having resources that can be used for attentional allocation and for revising beliefs and policies; and meaningfulness corresponds to being able to adopt revised understanding as a reality one can live within.

Individuals with high SOC retain flexibility in calibration and therefore tend to adapt by recalibrating even under stress. Individuals with low SOC show impaired calibration and are therefore more likely to develop more severe fixation under the same stress. For ESMR, this correspondence is more than metaphorical: in principle, it can be operationalized as ease of updating in response to corrective input and as reduced resistance to reality-grounded recalibration. It is best understood as a clinical bridge to the view of health as preserved recalibrability, not as the framework’s primary falsification target.

## 2 Where does calibration fail? The ESMR framework

### 2.1 The core problem: why do maladaptive responses persist?

Psychiatry and psychosomatic medicine repeatedly confront the same question: why do maladaptive responses persist even when individuals understand the cost of their own reactions?

When psychopathology is viewed as a failure of calibration, this question receives a process-level answer. Responses persist because calibration has become fixed at some layer. Is the body unable to exit a heightened bodily alarm-readiness state (E layer)? Does attentional allocation remain biased and fail to update (S layer)? Can beliefs be revised in principle but not in practice (M layer)? Does revision itself threaten self-coherence (R layer)?

Even for the same behavior, such as avoidance, the appropriate intervention differs fundamentally depending on which of these four layers sustains it. This is precisely the problem that existing symptom classifications cannot adequately capture.

### 2.2 What ESMR provides

**Figure 1:**
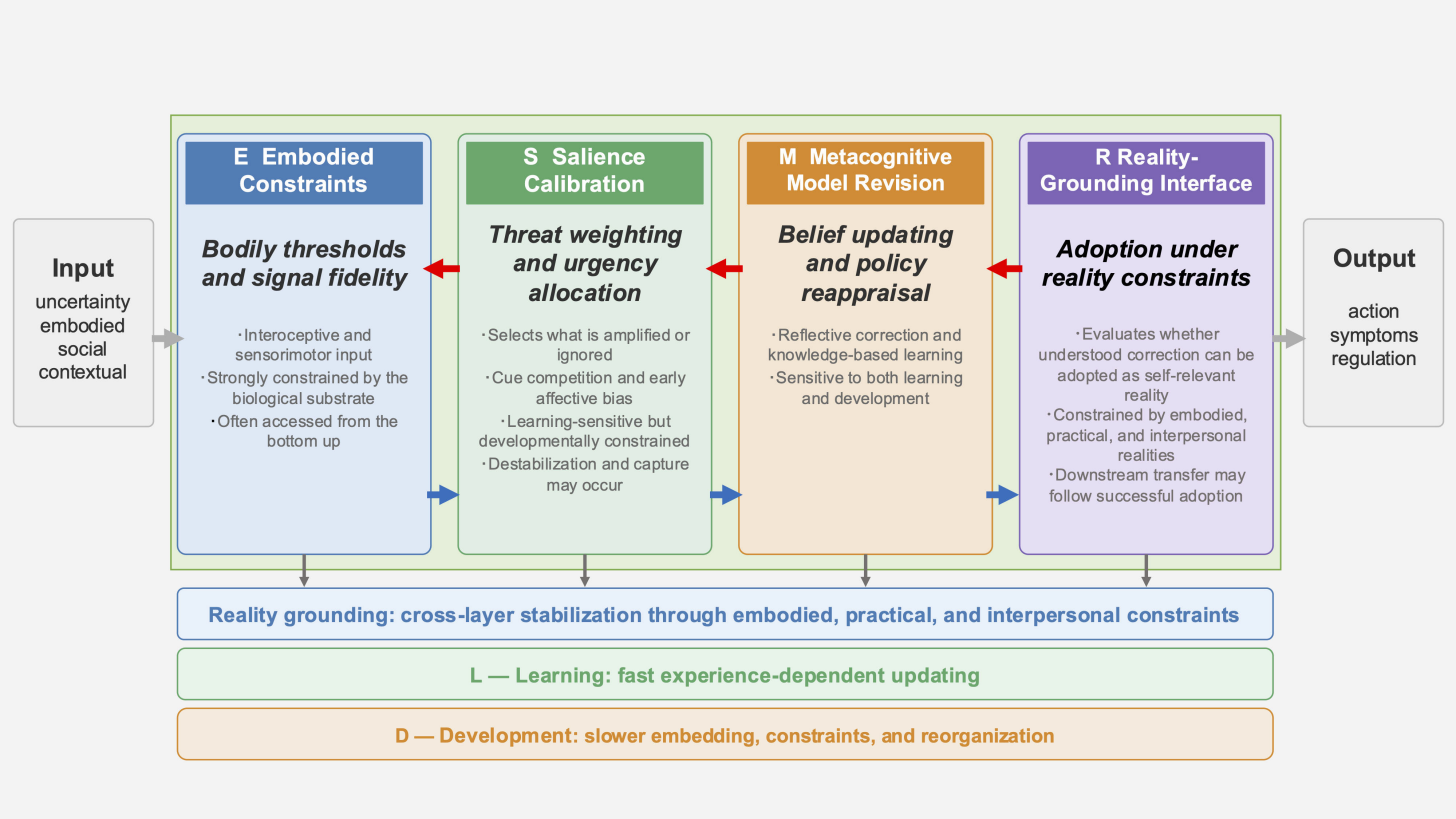
The layered ESMR calibration architecture under uncertainty. The model integrates stimulus-organism-response with learning (L) and development (D) and decomposes the organism into E (Embodied Constraints), S (Salience Calibration), M (Metacognitive Model Revision), and R (Reality-Grounding Interface). The arrows indicate bottom-up calibration flow and top-down prediction and policy constraints. At the R layer, information from E, S, and M is evaluated against embodied, practical, and interpersonal reality constraints to determine which correction candidates can remain personally tolerable enough to be adopted as self-relevant reality. At the behavioral level, successful or failed adoption at the R layer may appear as downstream transfer, or failure of transfer, from understood correction to enactment. The L/D axes denote not only the speed of change but also lifespan shifts in the biological and representational architecture that shape what forms of recalibration are possible.

**Figure 1** summarizes the layered ESMR architecture and its integration of stimulus-organism-response with learning and developmental timescales. The ESMR framework is a four-layer, two-timescale process framework for specifying where recalibration failure persists and at which timescale. It aims to move beyond current diagnostic systems by providing a comparable and falsifiable process map spanning psychosomatic conditions, psychiatric disorders, and psychosis-level phenomena. ESMR is not a unitary disease theory. It is a framework for comparing where recalibration becomes bottlenecked across heterogeneous clinical phenomena. Its originality lies in placing cross-signatures at the center of its predictions, distinguishing M-dominant generalized updating delay from R-dominant difficulty in adopting reality-grounded correction and linking these distinctions to layer-specific treatment targets.

The four functional layers indicate the level at which calibration occurs. The framework consists of E (Embodied Constraints), S (Salience Calibration), M (Metacognitive Model Revision), and R (Reality-Grounding Interface). R refers to the functional layer at which revised interpretations are evaluated against embodied, practical, and interpersonal constraints and either adopted or blocked as self-relevant reality, depending on whether correction remains tolerable under imperfect trial. In the present framework, downstream transfer into self-understanding, action, and acceptance is treated as an observable consequence of successful or failed adoption rather than as part of the R core itself. The detailed functions and operational distinctions of these layers are defined in the following section. ESMR is therefore proposed as a process framework for human organisms whose learning, representation, and recalibrability are shaped by embodiment, development, and aging, not as a disembodied computational template.

The two timescales indicate not only the speed and depth of change, but also the biological and developmental conditions under which different forms of recalibration become possible.

L axis (learning): short-term, experience-dependent recalibration operating within the currently available biological and representational architecture. L-level change may occur through bodily experience, relational experience, language-mediated correction, or explicit metacognitive revision, depending on developmental stage and current capacity.

D axis (development): slower changes in bodily, neural, representational, and self-regulatory architecture that alter what kinds of learning, revision, and fixation are possible at the L level in the first place. D therefore does not refer merely to the repeated embedding of prior L-level learning. It also includes maturational shifts, developmental reorganization, and aging-related changes that modify the quality, durability, and revisability of learning across the lifespan.

In the present framework, learning at the L-axis is not assumed to occur on a blank background. Recalibration at the fast timescale unfolds under historically shaped background conditions that influence how corrective input is tolerated, trusted, and stabilized. The D-axis therefore includes not only the slower embedding of repeated learning, but also slower changes in those background conditions themselves - that is, changes in the bodily, neural, representational, and self-regulatory architecture that shape what forms of learning remain possible.

One reason why the same intervention may work differently across childhood, adolescence, adulthood, and aging is that the architecture supporting recalibration itself changes over time. Early in development, learning is often mediated primarily through bodily and relational experience; with language development and later abstraction, symbolic interpretation and metacognitive revision become more available; and in later adulthood, accumulated response habits and reduced plasticity may make revision more effortful even when understanding remains intact. Repeated bias across the transition from L-to-D may progressively stabilize maladaptive solutions, setting the stage for the hyperstability discussed under Multiscale self-organization and hyperstability.

Development is not uniform across layers. At the E layer, primitive survival-oriented detection and regulatory priorities are available relatively early, whereas fine-grained sensory discrimination, bodily ambiguity resolution, and flexible calibration become more differentiated through maturation and may later decline with aging. Thus, the embodied architecture does not simply become stronger over time; rather, early-developing survival-oriented input systems coexist with later-developing refinement in bodily resolution and modulation (18–20).

At the E layer in particular, developmental change alters not only bodily thresholds and sensory resolution themselves, but also the informational quality on which later salience assignment, updating, and recalibration depend. Developmental differences in information-gathering capacity therefore become differences in learning conditions rather than mere differences in peripheral input (18–20).

By contrast, the M layer appears to undergo a disproportionate expansion in humans through language, abstraction, counterfactual capacity, and metacognitive revision. Adolescence may be a particularly important period in this trajectory, because higher-order reflective and control capacities continue to reorganize during this stage. This does not imply that the M layer is reducible to any single anatomical region. Rather, it suggests a biologically plausible developmental linkage between prolonged maturation of higher-order regulatory functions and the expansion of M-level revision capacity (18–20).

This expansion increases revisability, but it also enlarges the range of self-referential, evaluative, and coherence-threatening implications that corrective information can carry. On this view, one important source of specifically human psychopathology may lie in the growing mismatch between increasingly elaborated M-level revision and the more limited tolerability of R-level adoption under embodied, interpersonal, and self-coherence constraints.

A central claim of the framework is that M and R are operationally distinguishable. As defined in detail in the four-layer descriptions below, M concerns whether corrective information can be converted into updating, whereas R concerns whether an already understood, reality-grounded correction can remain personally tolerable under real-world constraints and therefore be lived as self-relevant reality. In M-dominant cases, updating is broadly delayed; in R-dominant cases, understanding may remain locally intact, yet adoption selectively stalls when correction carries self-coherence cost or when imperfect trial itself becomes difficult to tolerate.

This distinction is not intended as a mere relabeling of self-relevance weighting within existing predictive-processing hierarchies. Rather, the proposal is that M concerns the generation and conversion of corrective information into updating across conditions, whereas R concerns the selective adoption of already understood correction as self-relevant reality under reality constraints and self-coherence cost. On this view, R is introduced not as an additional generic precision term, but as a clinically differentiable bottleneck that predicts downstream transfer failure under self-relevant, reality-grounded conditions even when local revision remains possible.

The four-layer architecture is proposed as a pragmatic minimum decomposition rather than as a claim that no finer-grained subdivision is possible. Fewer layers would collapse clinically important distinctions, especially the differences between embodied constraint, salience-level appraisal of bodily and environmental signals, generalized failure of corrective updating, and selective failure to adopt already understood correction under reality constraints. More detailed subdivisions may become useful in future empirical work, but the present framework retains four layers because this is the smallest structure that preserves the main differential predictions targeted by the current falsification program.

At time *t*, the response can be schematically represented as follows:


$$Y_{t}=f\left(X_{t},E_{t},S_{t},M_{t},R_{t},L_{t},D_{t}\right)$$

*X_t_* denotes external stimulus input; E, S, M, and R denote the four functional layers; *L_t_* denotes the fast learning axis; and *D_t_* denotes slower developmental and structural constraints. Function f is treated not as a complete generative model, but as a clinically oriented decomposition for organizing identification, estimation, and intervention planning (see Minimal formalization and estimation strategy, below).

### 2.3 Differences from existing frameworks

RDoC organizes research around functional domains and constructs, whereas HiTOP emphasizes hierarchical symptom structure. In contrast, ESMR takes calibration dynamics - whether updating occurs, whether updating is blocked, and why the same corrective information has radically different effects across individuals - as its primary unit of analysis (21, 22).

The biopsychosocial model and the stress-vulnerability model provide useful overviews, but they remain relatively coarse with respect to comparing calibration dynamics across the range from psychosomatic conditions to psychosis-level phenomena, and to specifying at which layer such dynamics unfold. Whereas cognitive-behavioral models and metacognitive therapy describe loops of appraisal and action, ESMR explicitly identifies mechanism-level features that underlie those loops, including precision weighting, flexibility in policy selection, and reality grounding (23, 24).

Predictive processing and active inference provide powerful formalisms for describing how updating occurs, but they often treat the biological and developmental determinants of updateability itself as latent parameters, such as prior strength, precision weighting, learning rate, or controllability beliefs (1, 5, 6, 9). Although such quantities are in principle estimable from behavior, recent methodological work suggests that many computational measures still show limited psychometric stability, test-retest reliability, or interpretability as clinical individual-difference markers (25, 26). At least at the current stage, it is therefore difficult to treat such latent parameters as the primary currency of clinical theory. ESMR is proposed not in opposition to predictive processing or active inference, but as a clinically usable intermediate framework that translates updateability-related constraints into observable and intervention-relevant bottlenecks, including bodily capture, threat-framing sensitivity, delayed corrective updating, understanding-adoption gaps, and transfer failure. It therefore complements computational modeling by providing an observable, clinically explicit, and falsifiable description level that makes developmentally and clinically explicit what is often left abstract in computational formulations. This intermediate level is also consistent with the growing literature on psychometric and digital assessment of metacognition across the psychosis spectrum, which underscores the need for operational tools bridging theoretical constructs and observable clinical processes (25–28).

Related work in active inference has already addressed broad questions of psychopathology, body regulation, and interoception. Recent reviews have framed active inference as a translational program for psychology and psychiatry, while embodied accounts of homeostatic regulation and predictive-coding models of interoception have clarified how bodily signals participate in adaptive control and emotional experience (29–31). ESMR differs from these approaches in structural aim. Relative to broad active-inference syntheses of well-being, treatment, and psychopathology, it is narrower and more clinically discriminative: it is not a general theory of mind or treatment, but a pragmatic minimum decomposition built to distinguish where recalibration stalls. Relative to interoceptive and homeostatic hierarchy models, the E layer does not aim to model the full organization of bodily inference. It marks the embodied-constraint level within a wider four-layer architecture that separates bodily constraint (E) from salience-level appraisal (S), corrective updating (M), and self-relevant adoption (R). The distinctive prediction is therefore not simply that bodily prediction matters, but that M-dominant generalized updating delay and R-dominant selective failure to adopt already understood, reality-grounded correction should dissociate clinically and empirically.

The originality of ESMR lies in stating clearly that, for intervention planning, embodied safety constraints (E), salience redistribution (S), metacognitive revision (M), and restoration of reality grounding (R) should be distinguished as partially dissociable treatment targets. It also treats developmental constraints not as deterministic upper bounds, but as boundary conditions for recalibration (18–20).

A further point of departure is that ESMR treats human recalibration as biologically constrained across the lifespan. The framework does not reduce psychopathology to abstract information processing alone. It assumes that bodily maturation, sensory resolution, language acquisition, abstraction, metacognition, accumulated habit formation, and aging-related loss of plasticity all alter what forms of learning and correction are possible. ESMR is therefore a process framework for human organisms, not a computational template detached from biological development.

A practical limitation of broad biopsychosocial formulation is that, although it encourages multidimensional understanding, it often leaves unclear which domain should be prioritized for intervention in a given case. ESMR is intended to address this limitation not by replacing biopsychosocial reasoning, but by making its clinical use more explicit at the level of recalibration bottlenecks. By distinguishing whether the current constraint lies primarily in embodied alarm and input conditions, salience allocation, corrective updating, or the adoption of understood correction under reality constraints, the framework provides a process-based rationale for where intervention should begin and how sequencing should proceed. In this sense, the value of ESMR lies not only in broadening case formulation beyond symptom categories, but also in helping clinicians prioritize entry points for treatment.

## 3 ESMR in detail: a description of the four layers

In what follows, the definitions of E, S, M, and R - repeatedly referenced in later sections - are described in detail.

### 3.1 E: the embodied constraints layer

The E layer encodes interoceptive, autonomic, nociceptive, proprioceptive, and sensorimotor signals and specifies the bodily conditions under which signals can be detected, discriminated, amplified, or missed. It is shaped primarily by biological maturation and aging and, on the clinical timescale, functions both as an upper bound and as a set of developmental constraint conditions on bodily input fidelity and resolution.

Importantly, development at the E layer is non-uniform. Primitive survival-oriented detection and regulatory priorities are available relatively early, whereas finer sensory discrimination, bodily ambiguity resolution, and flexible modulation are refined more gradually through development and may later decline with aging. The amount and quality of bodily evidence available for later salience assignment therefore change across the lifespan.

The E layer determines not only how the body is stimulated but also the informational resolution available for learning. Differences in bodily signal quality, detectability, and ambiguity affect how experience is sampled in the first place, and therefore influence the quality, speed, and direction of later learning. When input is coarse, noisy, or easily amplified, subsequent salience assignment and belief revision may be built on unstable evidence. Conversely, when bodily input is more finely discriminated, later recalibration may proceed on a more reliable informational basis.

A further clinically important aspect of the E layer is that it may embody an already established baseline of bodily availability and bodily world-relation. In such cases, E-layer disturbance may appear not only as altered threshold, degraded resolution, or ambiguous input, but also as the loss of a previously available bodily condition. The clinical significance of losing vision, limb function, or bodily integrity may therefore differ fundamentally from congenital absence, because what is affected is not only current input, but the breakdown of an already formed embodied expectation of what the body can do or be.

In this framework, bodily capture refers to the involuntary incorporation of ambiguous bodily signals into threat-relevant interpretations, producing symptom amplification disproportionate to objective stimulus intensity.

Because recalibration may, at least in some cases, be facilitated from the bottom up by interventions at the level of bodily states, E may constitute a clinically accessible entry point for restoring recalibrability.

### 3.2 S: the salience calibration layer

The S layer governs salience allocation: it assigns relative significance to incoming bodily and environmental cues and determines which signals are treated as informative, urgent, threatening, safe, or otherwise worthy of learning. In computational terms, this includes precision weighting, cue competition, and the selective foregrounding of some inputs over others.

The S layer should not be reduced to emotion alone. Its primary role is not to generate explicit beliefs, but to shape which cues become dominant enough to bias affective response, orient attention, and constrain subsequent updating. Accordingly, S operates between embodied input conditions and higher-order revision: E shapes what can be registered, whereas S shapes what is treated as important.

Salience-weighted input can propagate along partially dissociable downstream pathways. In one route, it contributes directly to bodily-affective experience, such that the same bodily sensation may be felt immediately as alarm, urgency, or relief. In another route, it biases metacognitive revision by increasing the effective uptake of some cues and suppressing others. Thus, the S layer is the main locus at which danger, safety, and urgency are not yet fully conceptualized as explicit beliefs, but are already functionally shaping what the organism treats as reality-relevant.

The S layer therefore marks the transition from input quality to input meaning-weighting. If E determines the resolution of available evidence, S determines which portion of that evidence is treated as signal rather than noise and whether it is foregrounded in a threat-, safety-, or urgency-biased way.

Developmental disorders and stress-related states are treated here not simply as peripheral input problems, but often as disturbances in the weighting, amplification, and foregrounding of incoming cues. On this view, loss- or threat-framed contexts do not alter behavior by stipulation alone; rather, they shift the inferred relevance and expected cost of threat-congruent cues, thereby increasing the effective uptake of some forms of information while diminishing others (32, 33).

### 3.3 M: the metacognitive model revision layer

Clinically, the M layer becomes most visible when a person can recognize that a belief, bodily impression, or policy might be wrong, yet revision remains slow, inconsistent, or unable to convert disconfirming information into sufficient updating. A problem at the M layer does not simply mean that the person cannot notice an error. Rather, the problem is that a detected error does not translate into stable belief revision or policy change. Revision at the M layer operates most readily in domains for which at least a minimal framework of understanding or representation is already available. It includes perspective shifting between self- and non-self standpoints, comparative re-description of situations, and the critical holding of corrective alternatives long enough for updating to occur. The M layer becomes a bottleneck when a person can detect an error within that framework but cannot convert that detection into updating (24, 34, 35). This view is also consistent with emerging assessment work that treats metacognition as a clinically measurable process rather than a purely abstract construct (27, 28).

A further clinically important pattern arises when salience-weighted meanings generated at the S layer are taken up by M as if they were already epistemically adequate. In such cases, M does not simply fail to update after detecting an error; it may also fail to sufficiently re-describe or relativize threat-skewed meanings that have already been foregrounded by S, thereby allowing salience-biased interpretations to harden into apparently self-evident conclusions.

In humans, this revisional capacity is greatly expanded by language, abstraction, counterfactual simulation, and self-referential reflection, which increase both the range of possible corrections and the range of self-implicating consequences that those corrections can carry. Adolescence may represent a particularly important developmental window in this process, as higher-order reflective and regulatory capacities continue to reorganize during this period. This formulation is intended as a biologically plausible developmental linkage rather than as a strict anatomical identity claim (18–20).

Defensive reinterpretation may sometimes appear at this level because the same revisional machinery can be used to locally rationalize or reframe corrective input. Here, however, such operations are not treated as the primary purpose of M. Rather, they are often understood as self-protective uses of M-level revision recruited under higher self-coherence or self-existence cost at R.

### 3.4 R: the reality-grounding interface layer

The R layer is the functional interface at which signals from E and S, together with revisions generated in M, are evaluated against embodied, practical, and interpersonal reality constraints. Put simply, M asks whether a correction can be converted into updating, whereas R asks whether an already understood correction can be lived as self-relevant reality under real-world constraints. Its defining issue is whether correction remains tolerable when real-world trial involves temporary failure, partial incoherence, incomplete success, or evaluative risk. The R layer therefore governs the tolerability of corrigibility under conditions of self-existence threat (that is, threat to the self as a continuous and viable entity).

R does not primarily perform rational reevaluation; it functions as a bottleneck for personally tolerable adoption. Dysfunction therefore appears less as failure to understand corrective content than as failure to live it through imperfect trial. Adoption refers here to the mechanistic level of the R layer, whereas transfer refers to its later observable carry-over into behavior, self-understanding, and acceptance. Transfer is thus treated as a downstream indicator of stabilized adoption rather than as a separate core function. Importantly, the core issue at the R layer remains adoption under reality constraints rather than simple selection of the lowest-cost option. More specifically, the system must determine whether a correction can be adopted within a range of cost that remains survivable for the self. When reflective capacity, psychological reserve, and tolerability of failure are sufficient, individuals may accept higher immediate costs in order to pursue what is judged to be better, more reality-congruent, or more viable over the longer term.

The distinction from the M layer can be summarized as follows.

M-dominant impairment: the person can recognize that an interpretation may be inaccurate, but revision remains slow, inconsistent, or inefficient.

R-dominant impairment: the person can understand the content of a correction yet has difficulty living it as self-relevant reality when doing so requires tolerating temporary failure, threatened incoherence, or evaluative risk.

M asks how far beliefs and policies can be revised. R asks how readily revised understanding can remain personally tolerable under reality constraints and be carried into self-relevant output.

In M-dominant cases, updating delay tends to generalize across conditions with stronger and weaker reality-grounding support. In R-dominant cases, local understanding and partial revision may remain intact, yet adoption is selectively blocked when reality-grounded correction threatens self-coherence, belonging, social positioning, or the tolerability of being imperfect while trying.

Clinically, an M-layer bottleneck is exemplified when a person can acknowledge that a thought, impression, or policy may be inaccurate, yet still fails to revise it across situations. An R-layer bottleneck is exemplified when the person can say, in effect, ‘I know this is probably true, but I cannot live it as my reality,’ especially when adopting the correction implies possible failure, threatened incoherence, or exposure to negative evaluation.

The R layer is centered on adoption under reality constraints. Its core functions are (1) evaluating whether reality-grounded correction can be adopted as self-relevant reality under embodied, practical, and interpersonal constraints, and (2) maintaining that adoption through imperfect trial without immediate defensive collapse. Throughout this paper, real-world trial refers to the attempt to adopt and live a correction in actual life, whereas imperfect trial refers specifically to the fact that such enactment typically involves temporary failure, partial incoherence, or evaluative risk. Narrative integration and transfer are treated as downstream unfoldings rather than separate core layers. The self protected here includes not only momentary self-esteem, but also belonging, identity continuity, future outlook, and basic self-existence. In this paper, ‘payable without self-collapse’ refers to an anticipated cost range that does not imply collapse of biological viability, meaning, belonging, identity continuity, agency, or comparable forms of self-existence. The limiting condition at this layer is therefore not cost minimization per se, but whether the anticipated cost remains payable within that range.

This formulation does not turn R into a general theory of learning or social functioning. Bodily overload remains primarily an E-layer issue, threat foregrounding remains primarily an S-layer issue, and failure of correction-to-updating conversion remains primarily an M-layer issue. What R adds is a more precise account of whether understood correction can be lived through imperfect reality-bound trial without immediate defensive collapse.

This formulation yields a concrete and revisable clinical hypothesis: R-dominant difficulty should become most visible not in cognition alone, but when M-level understanding is relatively preserved while acting on that understanding requires tolerating non-catastrophic failure, temporary incoherence, or evaluative risk. The relevant tolerance is not only reflective or social-cognitive. It also depends on whether embodied feedback remains sufficiently safe that imperfect trial does not immediately recruit threat-dominant E/S responses or interpersonal destabilization. This tolerable cost range is likely to be strongly modulated by both learning-state conditions at L and more slowly embedded developmental organization at D.

Provisional evidence for R-dominant impairment requires more than distress, evaluation threat, or generalized avoidance alone. At a minimum, it requires that corrective content is locally comprehended, that broad updating failure is insufficient to explain the pattern, and that adoption or transfer deteriorates selectively when correction becomes both self-relevant and reality-constrained under coherence cost.

Human recalibration does not begin from a blank slate. It is constrained from the outset by embodied survival-relevant priors and by early learning about which bodily perturbations are tolerable versus catastrophic. On this view, later R-level adoption becomes possible only insofar as correction can be enacted under bodily and interpersonal conditions that keep imperfect trial within a tolerable range. The developmental expansion of M-level capacities is therefore double-edged: it enables comparative revision and social reflection, but it also increases the range of self-relevant implications that correction can carry, thereby making R-level adoption more vulnerable to self-coherence cost and evaluative threat.

**Table 1** summarizes the operational distinctions among E, S, M, and R. All subsequent task design and clinical translation are built on the distinctions summarized in **Table 1**.

**Table 1:** Discrimination matrix for the layers and timescales of ESMR.

Construct	Operational definition	Main observable indicators	Competing explanation	Discriminative criterion
E (embodied constraints)	Developmentally shaped bodily input conditions, including threshold, gain, fidelity, sensory resolution, bodily ambiguity, and information-gathering capacity; strongly constrained by maturation and aging and influential for later learning conditions.	Variability in autonomic arousal; bodily capture index; calibrated estimates of bodily thresholds	Mere general stress exposure or nonspecific symptom burden	The same pattern remains even after controlling for baseline stress/anxiety and introducing stimulus-intensity calibration and attentional-control trials.
S (salience calibration)	Salience allocation and precision weighting that assign relative significance to bodily and environmental cues, distinguish signal from noise, and foreground threat, safety, urgency, or relevance before explicit belief revision.	Threat-biased precision allocation; framing sensitivity; asymmetry in filtering/amplification	Trait negative affect or peripheral sensitivity alone	Predicts asymmetry in framing and filtering over and above the effects of calibrated bodily thresholds.
M (metacognitive model revision)	Perspective shifting and correction-to-updating conversion; markedly expanded in humans through language, abstraction, and self-referential reflective capacity.	Dynamic belief updating; policy reevaluation; insufficient relativization of salience-weighted interpretations	General cognitive ability	Specifically predicts globally slow updating under uncertainty across conditions with stronger and weaker reality-grounding support, and difficulty sufficiently relativizing salience-weighted meanings, rather than broad IQ proxy measures.
R (Reality-Grounding Interface)	A function that, under reality grounding, determines whether understood and reevaluated correction candidates can be adopted as self-relevant reality and remain personally tolerable under imperfect trial. Transfer into self-understanding, action, and acceptance is treated as a downstream consequence rather than as part of the R core itself. The limiting condition is not cost minimization alone, but whether adoption remains payable without self-collapse under embodied, practical, and interpersonal constraints.	Selective resistance to reality-grounded recalibration; large understanding-adoption gaps; low carry-over from understood correction into self-understanding, action, and acceptance; intolerance of imperfect trial	General irrationality, stable personality traits, social mistrust alone, or adjacent constructs such as fear of failure and intolerance of uncertainty treated in isolation	Beyond generalized delay in updating, revision can succeed under low-stakes or third-person conditions yet show a selective understanding-adoption dissociation when correction becomes personally relevant and reality-constrained.
L/D timescales	Fast learning (L) and developmental change (D). L denotes short-term recalibration operating within the currently available biological and representational architecture. D denotes slower changes in bodily, neural, representational, and self-regulatory architecture that alter what kinds of L-level learning, revision, and fixation are possible across the lifespan.	Learning gradients; persistence and recovery profiles; markers of developmental sensitivity; age-dependent shifts in learning quality and revisability	Mere chronicity	Shows layer-specific L/D weighting and developmental modulation of learning quality, rather than simple passage of time, repeated practice, or chronicity alone.

## 4 Reality grounding and the layer-specific understanding of defense mechanisms

### 4.1 What is reality grounding?

In ESMR, reality grounding refers to the degree to which an internal model remains constrained by bodily experience, practically testable environmental feedback, and interpersonally stabilized anchors. Bodily grounding is a narrower term for reliance on bodily sensation, agency, and interoceptive felt experience.

Within this framework, interpersonal stabilization is treated as an important component of reality grounding rather than as a separate master concept. It does not refer to simple conformity to majority opinion or uncritical acceptance of social norms. Rather, it denotes situations in which interpretations of self and world remain revisable through bodily evidence, practical consequences, and sufficiently stable interpersonal constraints.

Reality grounding and the R layer should be distinguished. Reality grounding describes a state: the degree to which the internal model remains constrained by embodied, practical, and interpersonal anchors. The R layer describes a function: whether such reality-constrained understanding can be adopted and lived as self-relevant reality without immediate collapse when real-world trial entails temporary failure, threatened incoherence, or evaluative risk. Its hallmark is therefore not simple irrationality or generalized rigidity, but selective difficulty in sustaining corrigibility under self-relevant conditions.

When reality grounding is weak, internally constructed representations of others and internal reference points become more vulnerable to threat-biased distortion. Variation at the R layer can therefore be understood as differences in how readily correction candidates are adopted under embodied, practical, and interpersonal constraints. This adoption threshold can be understood heuristically through reference dependence and loss aversion in prospect theory. However, the relevant process should not be reduced either to precision weighting or to choosing the lowest-cost option alone. In many cases, the key question is whether a correction can be adopted within a cost range that remains survivable for the self, given current reserve, time horizon, and tolerance of failure. When revised understanding is experienced as a loss of self-coherence, social position, or tolerability under imperfect trial, the threshold for adoption may rise even if the revision is objectively valid. Here, reference dependence is invoked only as a heuristic for thinking about the adoption threshold relative to self-coherence and social reference points, not to equate it with the framing effect addressed in A-1.

### 4.2 A layer-specific understanding of defense mechanisms

The same surface behavior, such as avoidance, may reflect rapid threat tagging in S, filtering of revision in M, or coherence-preserving resistance in R (36, 37).

At the S layer: rapid threat tagging, narrowing of attention, and overweighting of cues.

At the M layer: delayed incorporation of corrective evidence, unstable perspective shifting, and insufficient redescription or relativization of salience-weighted meanings, allowing threat-skewed interpretations to harden into apparently self-evident conclusions.

At the E layer, clinically significant disruption may arise not only from altered threshold, ambiguity, or amplification, but also from the loss of an already established embodied baseline, such that acquired loss is experienced as a breakdown of a previously available bodily world rather than as mere reduction of input.

At the R layer: even when the person understands corrective content, translating it into self-understanding, action, and acceptance may become difficult because doing so requires tolerating discontinuity, incomplete success, or possible failure in reality. When correction is experienced as a threat not only to coherence and belonging but also to the tolerability of being imperfect while trying, defensive solutions become more likely to dominate. In such cases, the system may preferentially adopt interpretations that preserve the self from immediate collapse, thereby drifting toward selective disregard of reality, attack on others, retreat into a private world, or other self-protective solutions (36, 37).

Especially at the R layer, repeated dependence on the same defensive solution can impede long-term recalibration and contribute to fixation across the L-to-D timescale. When maladaptive defenses persist, interpersonal relationships and social feedback can become harder-to-calibrate environments, and with chronicity they are more likely to consolidate into recognizable clinical pictures.

### 4.3 Human-specific vulnerability

The suffering generated by calibration failure is inseparable from the strengths of human sociality and reflective self-world modeling. Humans can flexibly adapt to others, norms, and changing environments. Yet precisely because of that capacity, transient bodily stress can be amplified through social reference points and self-world modeling into persistent, meaning-laden pathology. ESMR treats sociality not as the sole cause, but as a mechanism that can amplify meaning-laden stress (38, 39).

## 5 Fixation of calibration: a spectrum from health to psychosis

**Figure 2:**
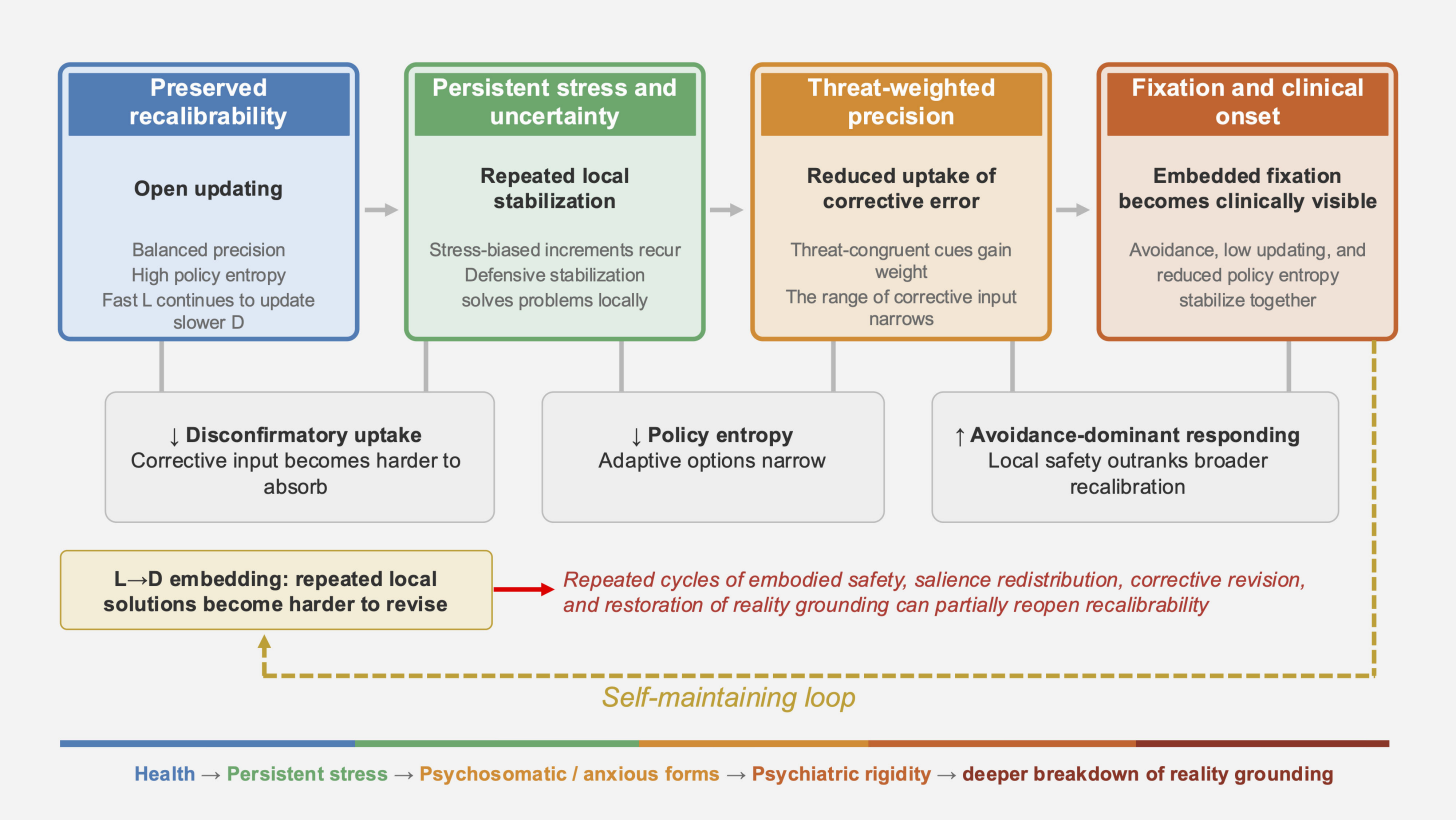
From recalibration to fixation. Onset is depicted not as a single event, but as a process in which calibration failure accumulates through L-to-D embedding and becomes clinically visible. Chronic uncertainty and threat-biased precision allocation may strengthen fixation through reduced uptake of disconfirming evidence, lower policy entropy, and increased avoidance-dominant responding. Conversely, repeated embodied safety, salience redistribution, corrective revision, and restoration of reality grounding may partially reopen recalibrability. The lower continuum schematically depicts possible clinical expressions of increasing fixation depth, from preserved recalibrability and persistent stress to psychosomatic/anxious forms, psychiatric rigidity, and deeper breakdown of reality grounding. This continuum is intended as a heuristic process map, not as a single nosological or deterministic disease progression.

**Figure 2** summarizes the process by which preserved recalibrability may shift toward clinically visible fixation through chronic uncertainty, threat-biased precision allocation, reduced disconfirmatory uptake, and L-to-D embedding.

### 5.1 Layer-specific fixation generates the spectrum

Calibration failure produces different clinical pictures depending on the layer at which it becomes fixed and the degree to which it does so. The key point is that these pictures are not sharply separated categories; rather, they can be understood as different expressions of the same calibration architecture (11, 12, 40–43).

When fixation centers on the E layer, bodily detection thresholds and input resolution may become distorted, and the calibration of interoceptive signals may become biased. The condition often appears as bodily symptoms, but its essence lies in the chronic accumulation of prediction error at the bodily level. For example, the same palpitations or fatigue may become fixed as low-threshold, ambiguously resolved, or chronically amplified bodily events, making it difficult for the organism to return to a more regulated baseline. When such bodily events are subsequently foregrounded as dangerous or urgent, that further meaning-weighting is treated in ESMR as a function of salience calibration at the S layer rather than of E alone. Psychosomatic conditions can often be understood as states in which unresolved tension, arousal, and action readiness are expressed through bodily channels when the person lacks sufficient means of symbolization, action, or relational adjustment to process the mismatch. This view is also consistent with evidence that symptom perception does not correspond one-to-one with physiological change, and that repetitive worry and threat anticipation can prolong stress-related physiological activation (44, 45). It also fits conceptualizations that regard persistent bodily symptoms not as mere absence of organic disease, but as products of interactions among prediction, attention, and stress physiology (46).

When fixation centers on the S layer, the allocation of what counts as important becomes fixed toward threat, and attentional allocation remains biased rather than updating. The same stimulus elicits disproportionate emotional responses. For example, ambiguous facial expressions in others or ambiguous bodily sensations are persistently tagged as threatening. Many forms of anxiety, phobia, and post-traumatic stress disorder are centered at this layer. Chronic bodily symptoms are also often organized as the coupling of bodily conditions in E and threat-biased salience allocation in S.

When fixation centers on the M layer, revision of beliefs and policies stalls; interpretation and action selection become rigid. A person may know what would be better yet still be unable to change. A person may realize that a thought might be distorted yet still be unable to revise it. In some cases, salience-weighted meanings that were foregrounded earlier remain insufficiently re-described or relativized, allowing threat-skewed interpretations to harden into apparently self-evident conclusions. For example, even in the face of disconfirming evidence, the person cannot update interpretations or action selection and repeatedly falls back on the same coping response. Depression, obsessive-compulsive phenomena, and some personality disorders include fixation at this level.

When fixation centers on the R layer, living revised understanding as output consistent with reality becomes difficult. The problem is not simply that the person holds an incorrect belief. Rather, revised understanding threatens self-continuity, belonging, agency, and the tolerability of imperfect trial, so corrective feedback is not readily adopted as self-relevant reality and therefore does not reliably carry over into self-understanding, action, and acceptance. For example, the person may understand something intellectually yet be unable to adopt that understanding as self-relevant reality and act on it. In M-dominant cases, updating tends to be globally slow across conditions with stronger and weaker reality-grounding support. In R-dominant cases, local understanding and partial revision may occur, yet corrections with stronger reality-grounding support become selectively difficult to adopt in everyday life when they carry a cost to self-coherence or make failure feel like direct self-negation. Importantly, the system may then default not simply to the least costly option in the abstract, but to what appears payable without collapse of self-existence under current L- and D-level constraints. When failure is experienced as direct negation of the self, avoidant choices - not acting in order not to fail - are easily reinforced. When self-negating ideation persistently undermines self-worth and agency, defenses become stronger and may appear as distortion of reality, treating events as if they had not happened, disconnection of memory, or retreat into a private world. Severe personality disorders, the psychosis spectrum, and dissociative disorders may involve this level of impairment.

### 5.2 Timescale: from acute to chronic

The depth of fixation also corresponds to the L and D timescales.

Fixation at the L-axis (learning) level allows for relatively rapid updating. With appropriate intervention, new learning can emerge and recalibration can recover. This level constitutes the therapeutic window.

Fixation at the D-axis (development) level reflects not only the structural embedding of repeated experience, but also developmental changes in the biological, representational, and self-regulatory architecture that constrain what forms of recalibration remain available. Change is difficult but not impossible. However, it requires a longer process involving repeated cycles of embodied safety, salience redistribution, corrective experience, and restoration of reality grounding, together with attention to the currently available developmental capacities through which new learning can actually be taken up. Developmentally embedded fixation does not imply therapeutic closure.

This temporal distinction also helps answer the clinical question of why the same intervention works for some people but not for others. If fixation remains at the L-axis level, a brief intervention may be effective. If fixation is embedded at the D-axis level, the first priority is to restore recalibrability at the current level of state and structure before attempting later-stage revision.

### 5.3 Clinical pictures are determined by combinations of layer and timescale

The claim is not that psychosomatic conditions and schizophrenia are the same disease. Failure of calibration of bodily signals and failure of reality-grounded adoption are different levels of dysfunction within the same predictive-processing architecture. This distinction is clinically important because symptom labels alone do not reveal where intervention should begin. Two people with anxiety may require entirely different treatment approaches if one is organized primarily around bodily hyperarousal in E, whereas the other is organized primarily around fixation of meaning in R.

## 6 Testable hypotheses and falsifiability

The central empirical prediction of ESMR is the presence of cross-signatures. Rather than a single main effect, the characteristic signature of ESMR is that response patterns differ systematically across layer-dominant profiles. Most clinical cases are mixed rather than pure single-layer profiles, and dominant bottlenecks should therefore not be treated as fixed person-level traits. The same individual may express different dominant bottlenecks across family, occupational, therapeutic, or peer contexts, depending on shifts in embodied alarm, salience bias, reality-grounding support, and self-coherence cost. The practical aim of the framework is thus not to assign a person to a context-invariant category, but to identify which bottleneck is currently most limiting recalibration under a given set of conditions and which manipulation is therefore most likely to shift response.

Even when two individuals show the same surface behavior, such as avoidance in response to threat, the framework predicts that they should respond differently across tasks if different layers are dominant.

E-dominant: high bodily ambiguity and bodily capture; strong response to bottom-up regulation; corrective revision becomes easier once arousal settles.

S-dominant: strong threat-framing sensitivity; reality-grounded recalibration remains relatively preserved when anchors are reliable.

M-dominant: corrective updating is slow despite preserved practical and interpersonal reality anchors; the person can recognize the possibility of error, yet updating does not proceed, and salience-weighted interpretations may remain insufficiently relativized.

R-dominant: selective resistance to adopting reality-grounded correction, despite relatively preserved local understanding, together with reinterpretation of corrective interpersonal evidence; the person may understand the correction intellectually yet still have difficulty adopting it as self-relevant reality, and the threshold for choosing actions rises especially for novel or unforeseeable behaviors.

A compact signature summary of the expected task-by-layer cross-signatures is presented in **Table 2**.

**Table 2:** Compact signature matrix (illustrative).

Profile	A-1 Framing	A-2 Updating	A-3 Reality-grounded recalibration	A-4 Bodily ambiguity/capture
E-dominant	Mild to moderate	Largely preserved once arousal settles	Relatively preserved once bodily alarm decreases	Bodily capture and threshold change are strongest
S-dominant	Threat-framing sensitivity is strongest	Variable; often context-biased	Often more flexible than in R-dominant cases	Amplification depends more on salience cues than on raw bodily intensity
M-dominant	Choice patterns may resemble those of S-dominant cases	Corrective updating is globally slow, confidence revision is weak across stronger and weaker reality-grounding conditions, and salience-weighted interpretations remain insufficiently relativized	Reality-grounded cues may be partly usable, and understanding-adoption gaps or self/third-person differences are not necessarily selectively maximal	High bodily capture is not necessarily present
R-dominant	May or may not show strong framing	Local understanding and partial updating may remain relatively intact under low-stakes conditions, but adoption as self-relevant reality is unstable.	Self-relevant adoption is selectively impaired, especially when correction is strongly reality-grounded and imperfect trial threatens coherence, belonging, or evaluative standing; downstream transfer may also deteriorate.	Bodily cues may be secondarily recruited to preserve coherence

### 6.1 Main hypotheses

H1 (primary hypothesis): compared with safety framing, loss/threat framing will reduce dynamic belief updating and increase avoidance and safety-policy selection. However, the magnitude and pattern of this effect will differ by layer-dominant profile rather than constituting a single undifferentiated clinical burden.

H2a (primary test of reality-grounding differentiation): corrective feedback supported by stronger versus weaker reality-grounding anchors is not functionally equivalent. Participants with stronger R-dominant fixation will be able to use weakly reality-grounded correction to some degree but will show disproportionately strong resistance when correction is more strongly reality-grounded and self-relevant.

H2b (exploratory mechanistic pathway): the effect predicted in H1 will be partially mediated by threat-biased precision weighting and reduced incorporation of disconfirming evidence.

H2c (exploratory moderation): lower defensive flexibility is predicted to amplify the association between loss framing and both reduced dynamic belief updating and increased selection of avoidant policies. This hypothesis is treated as auxiliary and is not part of the primary falsification set.

If H1 and H2a are treated as joint primary tests, family-wise error should be controlled using a preregistered procedure such as Holm correction (47, 48).

### 6.2 Design of the validation tasks (Set A)

Set A is designed for low-burden, reproducible, staged testing. In addition to three cognitive tasks, it includes one embodied/interoceptive extension (A-4).

Because the proposed layers are expected to be partially correlated and mutually reinforcing in practice, layer-dominant profiles are not intended to be defined *post hoc* by the same task outcomes that the framework is meant to explain. Rather, the empirical program is intended to use preregistered anchor indicators and competing models to distinguish shared severity or generalized distress from layer-weighted signatures. A critical step will therefore be to compare a general-severity or shared-arousal account against models that partition shared versus layer-specific variance. In practice, this could involve preregistered comparisons among (i) a general severity/arousal model, (ii) correlated latent-factor models, and (iii) a bottleneck model in which residual layer-weighted signatures add predictive value beyond shared variance. At the present stage, a pragmatic identification strategy would be to define provisional layer dominance using anchor indicators that are not identical to the primary explanatory outcomes: bodily ambiguity, bodily capture, and change under bottom-up regulation for E; framing sensitivity for S; generalized updating delay across stronger and weaker grounding conditions for M; and an understanding-adoption or understanding-transfer dissociation under self-relevant reality-grounded conditions for R.

A-1 Framing task: gain/loss or safety/threat framing under identical probabilities. Outcomes: choice rate, reaction time, and change in confidence. This task primarily probes precision bias and policy narrowing in the S layer (32, 33).

A-2 Repeated disconfirmation paradigm: trial-by-trial disconfirming evidence against an initial belief. Outcomes: updating slope, curvature, and confidence recalibration. This task primarily probes revisability at the M layer (24, 49).

A-3 Reality-grounded recalibration task (an R-related task family probing self-relevant adoption of correction): revision of decisions following corrective cues that vary in the degree of reality-grounding support. Stronger grounding is defined not as social consensus alone but as the joint presence of at least two of the following: (i) embodied or experiential plausibility, (ii) practically testable consequences, and (iii) interpersonally meaningful referential feedback that stabilizes mutual understanding. The task therefore probes whether participants can adopt correction as their own reality rather than merely repeat it.

The main discriminant aim is to distinguish R-dominant understanding-adoption gaps from broader alternatives such as social conformity, generalized avoidance, rejection sensitivity, or evaluation anxiety. Whereas those alternatives predict broader withdrawal or distortion under social threat, the ESMR prediction is selective degradation when correction must be adopted as self-relevant reality under stronger reality-grounding constraints.

For initial implementation, stronger designs should first anchor comprehension through paraphrase-based restatement, delayed recognition, or equivalent checks, and only then test adoption. A minimal paradigm could compare (a) simple factual correction, (b) correction plus practical confirmation, and (c) correction plus practical confirmation together with the other person’s explanation and subsequent reapproach. Presenting each in both third-person and self-relevant versions, while measuring understanding separately from reselection and policy change, should help distinguish self-relevant adoption difficulty from generalized updating delay.

Initial designs should preregister comprehension criteria, primary adoption outcomes, and a separate transfer index, while modeling baseline conformity tendency, generalized avoidance, perceived evaluation threat, rejection sensitivity, and subjective emotional intensity as competing predictors where feasible. For early validation, a staged sample spanning the nonclinical-to-clinical boundary is a realistic starting point.

A-4 Bodily ambiguity/bodily capture task (extension to psychosomatic conditions): ambiguous interoceptive or symptom-related cues are combined with attentional control conditions and intensity-calibrated trials. Outcome measures are symptom amplification, threshold change, and framing sensitivity. The key discriminative question is whether amplification tracks bodily threshold manipulation itself, or whether it tracks framing and attentional set more strongly.

**Figure 3:**
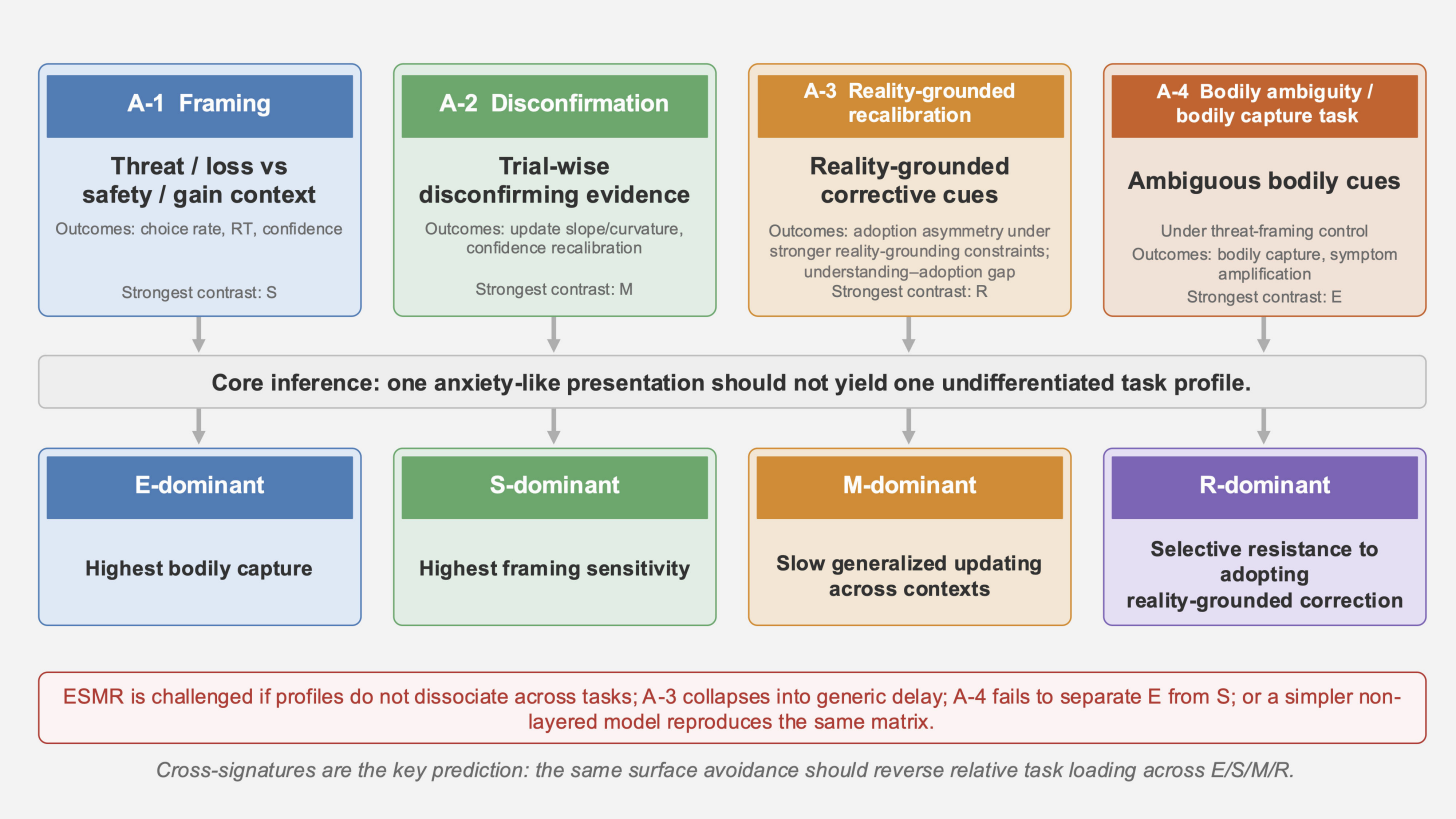
Predicted differential task signatures across provisional layer-dominant conditions in ESMR (Set A). The layer-dominant profiles shown here are provisional bottleneck conditions for comparative task prediction, not fixed person-level traits. A-1 (framing) primarily captures S-layer threat-framing sensitivity and policy narrowing. A-2 (disconfirmation) primarily captures generalized updating delay; in comparative analyses, M-dominant cases are expected to show such delay across both stronger and weaker reality-grounding conditions. A-3 (reality-grounded recalibration) probes selective difficulty in adopting already understood correction as self-relevant reality under stronger reality-grounding support. A-4 (bodily ambiguity/bodily capture) probes E-layer amplification under ambiguous bodily input. The distinctive prediction is not a single burden effect, but a cross-signature pattern across tasks.

Primary outcomes are: (i) dynamic belief updating, (ii) avoidance/safety preference, (iii) asymmetry across stronger versus weaker reality-grounding conditions, and (iv) amplification of bodily ambiguity and bodily capture. **Table 3** summarizes the task structure, core outcomes, predicted directions of Set A, and the mapping of each task onto specific layer-level predictions. **Figure 3** summarizes the differential task logic and the predicted cross-signatures.

**Table 3:** Overview of set A tasks.

Task	Core manipulation	Primary outcomes	Exploratory outcomes	Predicted direction
A-1 Framing task	Presents identical probabilities under gain/loss or safety/threat framing.	Choice rate; reaction time	Change in confidence	S-dominant cases should show the strongest threat-framing sensitivity, while reality-grounded recalibration remains relatively preserved.
A-2 Disconfirmatory updating task	Presents trial-by-trial disconfirming evidence against an initial belief.	Updating slope/curvature	Confidence recalibration	M-dominant cases should show globally slow corrective updating and policy switching across stronger and weaker reality-grounding conditions.
A-3 Reality-grounded recalibration task family	Manipulates the strength of reality-grounding support and self-relevance and then requires revised decision-making and output adoption.	Reselection rate; asymmetry across stronger versus weaker reality-grounding conditions; understanding-adoption gap	Confidence recalibration; reinterpretation of corrective cues; self/third-person difference; transfer from understanding to output	In R-dominant cases, even when participants can understand and repeat the corrective content, they should show the greatest difficulty in adopting self-relevant, reality-grounded correction as sustained self-understanding, action, and acceptance—especially when correction requires tolerating temporary failure, partial incoherence, or evaluative risk—and in transferring that adoption into everyday contexts. In M-dominant cases, updating should remain broadly slow across stronger and weaker reality-grounding conditions.
A-4 Bodily ambiguity/capture task	Presents ambiguous interoceptive or symptom-related cues under different threat-framing conditions, sometimes combined with high-arousal or misattribution conditions.	Symptom amplification and bodily capture	Change in body-related confidence	E-dominant cases should show the strongest bodily capture and threshold effects. In S-dominant cases, amplification should be more cue-driven; in M-dominant cases, bodily effects may be present but revisable; and in R-dominant cases, bodily cues may be secondarily incorporated into a broader narrative of reality mismatch.

### 6.3 Falsification thresholds and revision strategy

ESMR should be revised if two of its four core signatures repeatedly fail: (i) framing does not differentiate profiles; (ii) reality-grounded recalibration does not dissociate from weakly grounded correction; (iii) bodily ambiguity and bodily capture do not differentiate E-dominance from S-dominance after calibration control; or (iv) a simpler model reproduces the same cross-structure.

Revision can proceed in stages: refinement of measurement (Stage A), revision of architecture (Stage B), or restriction of scope (Stage C). The prediction-falsification matrix and the implications of related revisions are summarized in **Table 4**. **Table 4** therefore functions not only as a falsification matrix but also as a comparative-prediction matrix indicating where ESMR should outperform simpler alternatives.

**Table 4:** Falsifiability matrix and differential signatures of ESMR.

Prediction	Support pattern	Falsification pattern	Alternative model that may outperform ESMR	Implication for judgment
H1 Effect of framing on updating/avoidance	Loss/threat framing amplifies S-dominant profiles more than E-, M-, or R-dominant cases, even when surface avoidance is matched.	Framing fails to differentiate profiles after adequate power and control of confounds.	Arousal-only, mood-severity-only, or generic framing model	Weakens the S-layer claim and prompts review of boundaries.
H2a Reality-grounding differentiation test	Stronger reality-grounding support should reveal a cross-pattern. In R-dominant cases, understanding may remain under third-person or low-stakes conditions, but adoption should decline selectively when correction must be lived as self-relevant reality under stronger reality-grounding constraints. In M-dominant cases, broader updating delay should remain visible across both stronger and weaker reality-grounding conditions.	Differences between stronger and weaker reality-grounding conditions become negligible after adequate power and context control, and neither self/third-person differences nor understanding-adoption gaps emerge.	General distrust, broad social-sensitivity trait, or generalized updating-delay model	Narrows the R claim and prompts review of the M/R boundary.
H2b Precision pathway	Proxy indicators of threat precision partially mediate framing effects.	Behavioral effects appear without precision-related mediation.	Direct habit model without a precision stage	Refines the mechanism while retaining the behavioral claim.

## 7 Minimal formalization and estimation strategy

### 7.1 Notation and updating rules

The following formalization is intentionally schematic. It is included to organize estimation targets and falsification logic, not to claim a completed neurocomputational specification. To reduce ambiguity, observable output is denoted by *Y_t_*, reality grounding by the state variable *RG_t_*, self-coherence threat by *C_t_*, and R-layer gating dynamics by $G_{t}^{R}$. The layer labels E, S, M, and R refer to bottleneck locations rather than directly observed variables.

Belief updating (fast timescale):

$q_{t+1}=q_{t}+\;\alpha_{t}\omega_{t}\delta_{\text{eff},t}$

Here, *ω_t_* is assigned primarily by the S layer, *α_t_* is the effective learning rate, and *δ*_eff,_ *_t_* denotes the error that actually enters revision after higher-order gating. Under fixation, *δ*_eff,_ *_t_* may decrease selectively.

In plain terms, belief updating at the fast timescale is expected to slow when salience weighting, effective learning rate, or higher-order gating reduces the amount of corrective error that is allowed to count.

Learning increment:


$$\text{Δ}L_{t}\;=\;\alpha_{t}\omega_{t}\delta_{\text{eff},t}$$

Developmental updating (slow timescale):


$$D_{t+1}\;=D_{t}\;+\;\kappa P_{t}\underset{i\in \tau }{\sum }\text{Δ}L_{i}-\;\lambda {\text{Revision}}_{t}$$

Here, *P_t_* denotes current restructuring capacity—high during sensitive periods, and lower when the system is excessively stabilized or depleted. *κ* is a scaling parameter governing how strongly accumulated fast-timescale learning increments are carried into slower developmental embedding. Revision*_t_* denotes revision-related pressure acting on the developmental state at time *t*, that is, the extent to which ongoing correction, restructuring, or countervailing change opposes simple consolidation of prior learning. *λ* is the weighting parameter on that revision-related term and therefore determines how strongly such revision pressure modifies developmental embedding. Because bodily input fidelity, sensory resolution, and information-gathering conditions change with maturation and aging, E is the layer most directly shaped by D, and these changes alter the informational quality on which later salience assignment, learning, and recalibration depend. Here, *τ* denotes the set (time window) of learning indices whose increments Δ*L_i_* are accumulated into slower developmental embedding at time *t* (29–31).

In plain terms, developmental change alters how much restructuring remains possible, so the same corrective experience can have different effects depending on maturational stage, accumulated rigidity, age-related loss of plasticity, and the quality of information available for later learning.

### 7.2 Gate dynamics at the R layer

Let $G_{t}^{R}$ denote higher-order adoption-gating dynamics at the R layer, that is, the extent to which lower-layer corrective information is incorporated into self-relevant reorganization rather than blocked under self-coherence threat. The proposed gate is not intended to introduce a newly independent operator separate from hierarchical active inference. Rather, it is a clinical bottleneck representation of which correction candidates are treated as adoptable updates under self-coherence threat and reality grounding. It also reflects whether the anticipated cost of adoption falls within a range that remains survivable for the self under current L- and D-shaped conditions. The equations in this section serve as an illustrative scaffold rather than claims of strict neurocomputational identification of the R layer or a one-to-one mapping onto any single existing MDP parameterization; they are working hypotheses that place the relations to be empirically differentiated in compact form.

$G_{t}^{R}=\;\sigma \left(-aC_{t}+bRG_{t}-cD_{t}\right)$

Here, *C_t_* denotes current self-coherence threat, *RG_t_* denotes the current degree of reality grounding as a state variable, and *D_t_* denotes slow structural rigidity. By contrast, $G_{t}^{R}$ denotes the gating dynamics of the R layer that operate under those conditions. In interpretive terms, this gate can be thought of as approximating whether adoption remains payable without collapse of self-existence rather than as a simple least-cost selector.

In plain terms, R-layer gating is expected to weaken when self-coherence threat and structural rigidity are high and to strengthen when reality-grounding support remains sufficient for imperfect trial to stay tolerable and for the cost of adoption to remain survivable for the self.

The role of formalization in the present paper is intentionally limited. ESMR is proposed as a clinically oriented intermediate framework for organizing differential prediction, task construction, and intervention planning rather than as a fully specified computational model with uniquely identifiable latent parameters. The parameters *a*, *b*, and *c* are illustrative weighting terms included to indicate directional relations. The equation for $G_{t}^{R}$ is presented as a working-hypothesis representation to make the R-layer bottleneck visible. It is intended as a starting point for future embedding into richer hierarchical active inference models rather than as a completed formal account. The primary originality of the framework therefore lies not in the equations themselves, but in the claim that E-, S-, M-, and R-dominant bottlenecks should generate distinguishable cross-signatures across framing, disconfirmation, reality-grounded recalibration, and bodily ambiguity tasks.

## 8 Clinical translation

### 8.1 Process-level case formulation

The following section illustrates how ESMR can organize process-level case formulation across the spectrum from health to psychosomatic conditions, psychiatric disorders, and psychosis-level phenomena. These examples are intended as heuristic demonstrations of the framework’s comparative logic rather than treatment recommendations or claims about invariant disease progression.

Illustrative phenotype: interpersonal threat expectancy accompanied by fixed safety behaviors, bodily arousal, strong evaluative sensitivity to others’ responses, and a need to preserve a coherent self-other meaning.

E: developmentally shaped bodily input conditions, including threshold, sensory fidelity, bodily ambiguity, and the informational resolution available for later learning.

S: threat-biased salience weighting for ambiguous social signals.

M: weak revision of self-evaluative beliefs and insufficient relativization of salience-weighted interpretations despite locally available disconfirming evidence.

R: even when corrective understanding is partially achieved, that understanding is not readily adopted as output in self-understanding, interpersonal behavior, and acceptance.

A candidate sequence of intervention begins at the point where the current bottleneck can be modified most safely and productively. The basic flow is stabilization of E through embodied regulation (breathing, muscle relaxation, posture, movement, touch, and related techniques), recalibration of S through redistribution of salience, threat weighting, and affective gain, strengthening of M through corrective learning and metacognitive revision, and support for R through repeated experiences of reality grounding and restored interpersonal or practical alignment. If high-demand cognitive restructuring is introduced prematurely, it may instead amplify defensive activation at the R layer in cases with high self-coherence threat. A staged updating process built on small and safe experiences of success is therefore preferable, partly because it can expand the range of costs that remain payable without self-collapse.

Illustrative vignette 1 (E-dominant): a patient with recurrent dizziness, chest discomfort, and amplified bodily capture under ambiguity. The patient shows marked improvement after paced breathing and posture-based regulation. Once arousal settles, incorporation of corrective interpretation remains relatively preserved.

Illustrative vignette 2 (S/M-dominant): a patient with fear of negative social evaluation. The patient shows strong threat-framing sensitivity and slow confidence revision yet remains capable of responding to corrective feedback and guided reevaluation when salience competition is reduced.

Illustrative vignette 3 (R-dominant): a patient organized around shame, exclusion, and unreality. The patient can recognize corrective input verbally yet cannot stably adopt that content as output in self-understanding or interpersonal behavior and persistently reinterprets socially stabilizing feedback.

### 8.2 A practical measurement map for outpatient care

Before formal experimental differentiation, ESMR is intended to function as a clinic-friendly observational map. Its immediate value lies in helping clinicians distinguish whether the current bottleneck is primarily expressed as bodily capture, threat-weighted salience allocation, delayed corrective updating, or failure to adopt already understood correction under reality constraints.

E: concise indicators of autonomic arousal, bodily discomfort, symptom amplification under ambiguity, change under bottom-up regulation, and gross variation in bodily uncertainty or input clarity.

S: framing sensitivity, threat-weighted confidence allocation, and filtering/amplification patterns.

M: indices of corrective updating slope, confidence recalibration, metacognitive monitoring, and insufficient revision or relativization of salience-weighted interpretations (24).

R: selective resistance to reality-grounded correction, reinterpretation of consensus cues, poor practical transfer despite preserved verbal understanding, and narrowing of what costs can be borne without perceived self-collapse. At this layer, acceptance and commitment therapy can be mapped hypothetically onto processes that reduce experiential avoidance through value-based action, and schema therapy can be mapped hypothetically onto processes that may lower the adoption threshold through mode work and limited reparenting (50, 51).

Indicators of successful recalibration are not limited to symptom reduction. Reduced bodily capture, accelerated corrective updating, improved translation of revised beliefs into behavior, and restored participation in interpersonal and role contexts can all serve as provisional markers of greater flexibility at the R layer.

A minimal outpatient workflow can be implemented across three visits.

Baseline: identify the dominant bottleneck (E: high bodily ambiguity or bodily capture; S: threat overweighting; M: low corrective updating or insufficient relativization of salience-weighted meaning; R: self-social or reality mismatch). In the outpatient setting, a simple practical clue for roughly distinguishing M-dominance from R-dominance is to determine whether the person can understand the possibility of error yet cannot revise the thought or adequately relativize a salience-weighted interpretation, or instead can understand the validity of a correction but feels that adopting it as self-relevant reality would destabilize the self and therefore cannot translate it into output. This distinction is the minimal outpatient implementation of the operational difference summarized in **Table 1**.

Micro-intervention: apply an intervention matched to the bottleneck (E: arousal regulation and reduction of bodily uncertainty; S: precision redistribution or reframing; M: corrective learning tasks; R: repeated reality-grounding exercises).

Follow-up: examine whether the bottleneck has shifted and, if change is limited, revise the layer-based formulation.

The central prediction is sequence dependence: when the threat state at the E and S layers remains high, later corrective learning may be understood verbally but still fail to consolidate or generalize. Conversely, early recovery of bodily safety and attentional flexibility should increase the likelihood that later updating and adoption will take hold.

Existing interventions can be located within this map. Body-focused methods, including breathing-, posture-, movement-, and touch-oriented approaches, can be mapped most closely onto E (52). Attention training, restructuring of exposure, and redistribution of salience can be mapped most closely onto S. CBT-style corrective learning and metacognitive therapy can be mapped most closely onto M (53, 54), whereas interventions that support reality-grounded meaning-making and interpersonal validation can be mapped most closely onto R. These mappings are heuristic and are intended to guide hypothesis generation rather than to imply established treatment specificity.

## 9 Discussion

### 9.1 Theoretical implications

The major theoretical claim of ESMR is that partially shared calibration dynamics can be compared across stress responses, psychosomatic dysfunction, psychiatric syndromes, and psychosis-level disturbances within a single process framework. This is a comparative process claim, not a nosological claim that these conditions are the same disorder or differ only in severity.

The framework therefore supplements rather than abolishes descriptive diagnosis. Its added value is a treatment-oriented process map of where recalibration becomes bottlenecked and what kind of correction remains possible at a given time.

The distinction between M and R is central to the framework. M concerns whether corrective information can be converted into updating, whereas R concerns whether an already understood correction can be adopted and sustained as self-relevant reality when doing so requires tolerating temporary failure, threatened incoherence, or evaluative risk. The claim is not that M and R are fully independent faculties but that they create different bottlenecks, intervention sensitivities, and observable failure patterns.

A further implication of ESMR is that psychopathology unfolds against an asymmetrical developmental architecture. The E layer is strongly shaped by biological maturation from the outset, whereas the M layer expands disproportionately in humans through language, abstraction, counterfactual capacity, and metacognitive revision. This expansion increases revisability but also enlarges the range of self-referential and coherence-threatening meanings that correction can carry. One important source of human psychopathology may therefore lie in the tension between increasingly elaborated M-level revision and the more limited adoptability of correction at the R layer under embodied, interpersonal, and self-coherence constraints.

ESMR is thus best read as a biologically and socially situated process framework for human recalibration, rather than as a general theory of mind or a purely computational account. Its primary aim is narrower and more practical: to clarify why understood correction is sometimes updated, sometimes defensively reworked, and sometimes not livable as self-relevant reality at all.

A practically immediate first test of this observable-level claim is whether trained raters can achieve acceptable agreement when applying layer-based formulations to standardized vignettes or repeated outpatient observations. Before more demanding task-based adjudication, such Stage 0 work can test whether the proposed bottlenecks are clinically trackable in a reproducible way.

### 9.2 Multiscale self-organization and hyperstability

Hyperstability, introduced here as a derived dynamic consequence of repeated L-to-D embedding, refers to a state in which repeated biased increments in prior rigidity, elevated threat precision, and reduced policy entropy make revision progressively more difficult.

The ESMR layers are better understood as a recursive control architecture than as independent boxes. Bottom-up calibration carries bodily signals from E into salience allocation in S, shapes revision in M, and constrains enactment in R. Conversely, output tendencies stabilized at R feed back into S by biasing which mismatches are treated as dangerous or tolerable, thereby shaping the next round of attention, prediction, and policy selection.

These interactions are not assumed to be fully symmetrical across layers. Salience allocation influences revision, but repeated M-level reinterpretation can also reshape later salience patterns by changing which cues are anticipated, foregrounded, or treated as self-relevant. Likewise, repeated conflict between M-level revision and R-level adoption may further bias what becomes livable, believable, or defensively resisted over time. The E layer is likely to remain the most strongly constrained by biological and developmental conditions, although higher-layer patterns may still alter its effective thresholds, bodily ambiguity, or alarm-readiness gradually through repeated embedding across the L-to-D timescale.

When such bias persists across the L-to-D timescale, the system may settle into a pathologically narrow dynamic equilibrium: locally stabilizing, but progressively less revisable. Psychopathology, on this view, is not mere loss of function in the abstract, but fixation of once-functional response tendencies into increasingly rigid equilibria. This way of framing rigidity is also compatible with related work on allostatic load, attachment-based regulation, predictive dissonance, and hierarchical precision control (55–62).

### 9.3 Clinical implications

Clinically, ESMR suggests beginning with the layer that offers the safest and most effective entry point for recalibration, rather than assuming that insight-oriented work should always come first.

Therapeutic learning is state- and development-dependent. When bodily dysregulation or heightened bodily alarm-readiness in E and threat precision in S remain high, new information may be verbally grasped yet fail to be retained, trusted, or generalized. Even when revision is possible at M, adoption at R may still fail if the cost of living the correction remains self-threatening.

### 9.4 Limits and next steps

ESMR remains a revisable framework rather than a completed model. It does not yet provide model inversion, definitive layer boundaries, or a culturally invariant account of belonging, evaluative threat, and interpersonal reality stabilization. At this stage, the framework should therefore be judged by the clinical and empirical usefulness of its differential predictions. Several conditions that likely shape recalibration—including start conditions for correction, format-dependent uptake of corrective input, maintenance of change, and post-correction social re-entry—require higher-resolution treatment beyond the scope of the present paper.

### 9.5 A staged empirical roadmap

As a preliminary feasibility check (Stage 0; see Theoretical implications), the inter-rater agreement of layer-based formulations can be evaluated using standardized vignettes or repeated outpatient observations. The cumulative empirical roadmap can remain simple. First, test whether framing sensitivity, updating delay, reality-grounding asymmetry, and bodily ambiguity/capture dissociate in the expected directions. Then examine whether intervention response also differs by layer-dominant profile.

D-level fixation does not imply therapeutic closure. It indicates that biased response patterns have been embedded into the organism’s developmental organization and usually require longer, repeated recalibration cycles to loosen.

Taken together, ESMR defines health not as the absence of symptoms but as the preservation of recalibrability. Its strongest prediction is not a single burden factor, but cross-signatures: S-dominant cases should show high framing sensitivity, M-dominant cases generalized updating delay, R-dominant cases selective impairment in self-relevant adoption under stronger reality grounding, and E-dominant cases bodily ambiguity and bodily capture.

The practical value of the model lies in specifying where recalibration becomes bottlenecked, where adaptive buffering begins to fail, and how intervention can be matched to the biologically and developmentally available mode of correction. The capacity to tolerate small failure as part of correction is therefore not peripheral, but one of the core conditions for both clinical change and durable adaptation.

## Data Availability

The original contributions presented in the study are included in the article/supplementary material. Further inquiries can be directed to the corresponding author.
